# SARS-CoV-2 Infection Risk Among Active Duty Military Members Deployed to a Field Hospital — New York City, April 2020

**DOI:** 10.15585/mmwr.mm7009a3

**Published:** 2021-03-05

**Authors:** Guy T. Clifton, Rituparna Pati, Florian Krammer, Eric D. Laing, Christopher C. Broder, Damodara R. Mendu, Mark P. Simons, Hua-Wei Chen, Victor A. Sugiharto, Anthony D Kang, Daniel Stadlbauer, Kathleen P. Pratt, Bradley C. Bandera, Darron K. Fritz, Eugene V. Millar, Timothy H. Burgess, Kevin K. Chung

**Affiliations:** ^1^Department of Surgery, Brooke Army Medical Center, Fort Sam Houston, Texas; ^2^CDC COVID-19 Response Team; ^3^Department of Microbiology, Icahn School of Medicine at Mt. Sinai, New York, New York; ^4^Department of Microbiology and Immunology, Uniformed Services University of Health Science, Bethesda, Maryland; ^5^Department of Pathology, Molecular, and Cell Based Medicine, Icahn School of Medicine at Mt. Sinai, New York, New York; ^6^Naval Infectious Diseases Diagnostic Laboratory, Naval Medical Research Center, Silver Spring, Maryland; ^7^Infectious Disease Clinical Research Program, Department of Preventive Medicine and Biostatistics, Uniformed Services University of the Health Sciences, Bethesda, Maryland; ^8^Henry M. Jackson Foundation for the Advancement of Military Medicine, Bethesda, Maryland; ^9^Department of Pathology and Ancillary Laboratory Services, Carl R. Darnall Medical Center, Fort Hood, Texas; ^10^Department of Medicine, Uniformed Services University of Health Science, Bethesda, Maryland; ^11^Department of Surgery, Eisenhower Army Medical Center, Augusta, Georgia; ^12^Department of Emergency Medicine, Carl R. Darnall Medical Center, Fort Hood, Texas.

Protecting health care workers from COVID-19 remains a priority during the ongoing pandemic. Accurate assessment of the risk for infection among health care workers is important in determining the effectiveness of infection control plans. In late March 2020, a total of 591 U.S. Army personnel from three units were deployed from areas in which COVID-19 incidence was low to the Javits New York Medical Station (JMS), a 452-bed Federal Emergency Management Agency Federal Medical Station in New York City (NYC), to provide care to COVID-19 patients. Army personnel followed a rigorous infection control plan and remained largely isolated from the surrounding community while in NYC. During April 3–25, a total of 1,095 COVID-19 patients were admitted from NYC area hospitals to the JMS ward or intensive care unit (ICU). A cross-sectional study of the prevalence of SARS-CoV-2 infection among 336 active duty soldiers enrolled in a prevalence study identified an infection rate of 1.7% overall and 0.9% in the 223 (66.4%) enrolled soldiers who provided direct care to COVID-19 patients. A well-designed and well-implemented infection control plan can mitigate the risk for SARS-CoV-2, the virus that causes COVID-19, infection in health care settings, including nontraditional settings.

All patients transferred to JMS had received a positive SARS-CoV-2 polymerase chain reaction (PCR) test result or a clinical diagnosis of COVID-19 when evaluated at the transferring hospital, were clinically stable or improving, and did not have other complex medical conditions. Upon arrival, patients were admitted to a 452-bed patient care area converted from an exhibition space. The ventilation in the JMS patient care area was adjusted to create a negative pressure differential related to all other JMS spaces. Beds were contained within 8-foot square pods separated by temporary dividers with open ceilings and cloth doorways, and pods were supplied with oxygen by concentrators or portable tanks. Patients whose clinical condition deteriorated after admission were transferred to the ICU within JMS or to a local hospital.

Active duty soldiers from three army units, the 9th Hospital Center (Fort Hood, Texas), 531st Hospital Center (Fort Campbell, Kentucky), and the 44th Medical Brigade (Fort Bragg, North Carolina), were deployed to JMS to care for COVID-19 patients. These soldiers were not routinely tested for SARS-CoV-2 before deployment to NYC, and none had previously received a positive SARS-CoV-2 test result.

A multidisciplinary team of infection control specialists proactively designed and implemented infection control procedures to protect health care personnel. All personnel were screened for COVID-19–associated symptoms and fever upon each entry to JMS. The patient care area had one entry point for health care personnel where personal protective equipment (PPE) donning was continually observed, with assistance provided. All personnel working within 6 ft of COVID-19 patients were fit-tested and wore N95 respirators, eye protection, gloves, disposable gowns, and single-use scrubs. All personnel were required to completely doff PPE, with assistance, at the doffing station before exiting the patient care area for breaks and were required to repeat the observed donning process to reenter.

Military personnel were the sole occupants of local hotels and were housed in single-occupancy rooms. The military chain of command encouraged deployed personnel to stay in their hotel rooms as much as possible when off duty; meals were available at JMS, through food delivery services, or by take-out from restaurants within short walking distances of the hotels. The chain of command enforced mask wearing and physical distancing at all times. Personnel were placed in command-directed isolation if they reported experiencing any COVID-19–associated symptoms.

To assess the prevalence of SARS-CoV-2 infection among active duty soldiers deployed to JMS, researchers recruited soldiers for the study in late April. Among 591 eligible soldiers who deployed to NYC beginning on March 24, a total of 336 (56.8%) consented to participate in the prevalence study during April 28–30. Among the enrolled soldiers, 298 (88.7%) originated from an area where the 3-day average COVID-19 incidence was <10 cases per 100,000 persons; in contrast, the 3-day average incidence in NYC during March 30–April 1, 2020, was 519 per 100,000.* All enrolled soldiers completed an anonymous study questionnaire that asked about demographic characteristics, duties at JMS, and history of COVID-19 symptoms, isolation, and testing. During April 28–30, 2020, nasopharyngeal swabs were collected from all enrolled participants and were tested using the TaqPathTM COVID-19 Combo Kit (Thermo Fisher Scientific).^†^ All participants also received enzyme-linked immunosorbent assay (ELISA) immunoglobulin G (IgG) and multiplex microsphere-based immunoassay (MMIA) IgG and immunoglobulin M (IgM) testing. Although not authorized for clinical diagnostic purposes at the time, MMIA can test simultaneously for antibodies to multiple antigens using an extremely small sample volume, in this case, <2 *μ*L. SARS-CoV-2 antibody presence and titer were ascertained by using ELISA and MMIA (*1*).^§^

Enrolled soldiers were considered to have been infected if SARS-CoV-2 viral RNA was detected on nasopharyngeal PCR tests or if SARS-CoV-2 IgG antibodies were detected by ELISA. MMIA serologic testing was used to improve the sensitivity of antibody detection. Medians were compared by Wilcoxon-rank sum test using SPSS Statistics (version 22.0; IBM).

This study was approved by the Uniformed Services University Institutional Review Board. The activities were reviewed by CDC and were conducted consistent with applicable federal law and CDC policy.^¶^

Among the 336 soldiers who participated in the study, 304 (90.5%) had arrived in NYC before the first COVID-19 patient was admitted to JMS (Figure). During April 3–25, a total of 1,095 COVID-19 patients were admitted to JMS. Throughout that time, 77 (13.0%) of 591 soldiers were tested for SARS-CoV-2 by PCR because of reported COVID-19–compatible symptoms, including four who received positive SARS-CoV-2 PCR test results. Among the 336 soldiers enrolled in the study, 45 (13.3%) were tested because of symptoms; two had a positive SARS-CoV-2 PCR result. 

**FIGURE:**
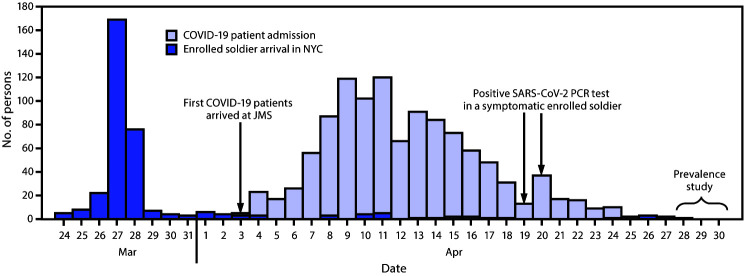
Admission of COVID-19 patients, arrival of deployed U.S. Army personnel enrolled in prevalence study, and dates of positive SARS-CoV-2 test results — Javits New York Medical Station (JMS), New York City (NYC), March 24–April 30, 2020 **Abbreviation:** PCR = polymerase chain reaction.

The 336 solders enrolled in the SARS-CoV-2 prevalence study included 100 registered nurses (29.7%), 99 medical support staff members (27.9%), 25 physicians and physician assistants (7.4%), and 117 other staff members (34.7%) (Table 1). Direct contact with COVID-19 patients was reported by 223 (66.4%) enrolled soldiers, for a self-estimated median of 240 hours at the time of enrollment and testing.

**TABLE 1:** Characteristics of active duty military members enrolled in a cross-sectional prevalence study of SARS-CoV-2 infection — Javits New York Medical Station (JMS), New York City (NYC), April 2020

Characteristic	No. (column %) of participants	
	All (N = 336)	SARS-CoV-2–positive (n = 6)
**Median age, yrs (IQR)**	**32 (25.3–40.0)**	22 (20.3–23.0)
**Sex**		
Female	**135 (40.2)**	2 (33.3)
Male	**201 (59.8)**	4 (66.7)
**Professional role**		
Registered nurse	**100 (29.7)**	1 (16.7)
Medical support*	**94 (27.9)**	1 (16.7)
Physician/Physician assistant	**25 (7.4)**	0 (—)
Other	**117 (34.7)**	4 (66.7)
**Median no. of days in NYC (IQR)**	**31 (30.0–32.0)**	31 (31.0–31.8)
**Travel within 2 wks before arrival**	**27 (8.0)**	0 (—)
**Potential exposure risks**		
Directly cared for COVID-19 patients	**223 (66.4)**	2 (33.3)
Median number of patient-care hours (IQR)^†^	**240 (176.0–288.0)**	264 (228.0–300.0)
Performed aerosol-generating procedures	**26 (7.7)**	0 (—)
Reported break in PPE	**36 (10.7)**	1 (16.7)
**Symptoms and isolation**		
Reported potential symptoms	**133 (39.6)**	4 (66.7)
Isolated for COVID-19–associated symptoms	**52 (15.5)**	2 (33.3)
Median days isolated (IQR)	**7 (7.0–7.0)^§^**	10.5 (10.3–11.0)
**Symptoms while in NYC, reported at enrollment, no. (%)**		
Fever	**16 (4.8)**	2 (33.3)
Cough	**32 (9.5) **	2 (33.3)
Shortness of breath	**14 (4.2)**	1 (16.7)
Diarrhea	**55 (16.4)**	1 (16.7)
Anosmia	**7 (2.1)**	2 (33.3)
Sore throat	**52 (15.5)**	3 (50.0)
Rhinorrhea	**81 (24.1)**	3 (50.0)
**Median duration of symptoms, when present, days (IQR)**		
Fever	**2 (2.0–3.3)**	3.5 (3.3–3.8)
Cough	**4 (2.0–7.0)**	8 (7.5–8.5)
Shortness of breath	**5 (2.0–6.0)**	8 (—)
Diarrhea	**2 (1.0–4.0)**	1 (—)
Anosmia	**2 (2.0–3.0)**	4 (4.0–4.0)
Sore throat	**3 (2.0–4.0)**	3 (3.0–6.0)
Rhinorrhea	**4.5 (2.0–7.0)**	3 (3.0–5.0)

**Abbreviations:** IQR = interquartile range; PPE = personal protective equipment.

* Medical support includes licensed practical nurses, medics, therapists, and other specialists who typically interact with patients.

^† ^Patient-care hours for those who directly cared for COVID-19 patients, determined by self-reported hours/week caring for patients and the time from arrival to NYC or the first admission of COVID-19 patients to JMS, whichever was later.

^§^ Because the majority of persons were isolated for 7 days, the 25th and 75th percentiles are both 7 days, as is the median.

During the SARS-CoV-2 prevalence study, six of 336 (1.7%) soldiers received a positive SARS-CoV-2 test result, either by nasopharyngeal swab PCR (two), ELISA (five), or both (one) (Table 2). The five soldiers with positive IgG test results had titers of 1:80 (three), 1:160 (one), and 1:2,880 (one). Two (0.6%) soldiers had detectable IgG antibodies to the spike protein and receptor binding domain by MMIA testing; both soldiers also had positive ELISA test results (titers = 1:160 and 1:2880). The three soldiers with ELISA titers of 1:80 had negative MMIA results. Both soldiers with positive MMIA IgG results also had detectable IgM to SARS-CoV-2 spike protein; all other IgM results were negative.

**TABLE 2:** Molecular test and serologic assay results among soldiers with positive SARS-CoV-2 test results (N = 6) — Javits New York Medical Station, New York City, April 2020

Soldier	SARS-CoV-2 test results				
	PCR	ELISA IgG	Titer	MMIA IgG	MMIA IgM
A	Pos	Pos	1:160	Pos	Pos
B	Neg	Pos	1:2,880	Pos	Pos
C	Neg	Pos	1:80	Neg	Neg
D	Neg	Pos	1:80	Neg	Neg
E	Neg	Pos	1:80	Neg	Neg
F	Pos	Neg	N/A	Neg	Neg

**Abbreviations:** ELISA = enzyme-linked immunosorbent assay; IgG = immunoglobulin G; IgM = immunoglobulin M; MMIA = multiplex microsphere-based immunoassay; N/A = not available; Neg = negative; PCR = polymerase chain reaction; Pos = positive.

The median age of the soldiers with positive SARS-CoV-2 test results (22 years) was significantly younger than that of all enrolled soldiers (32 years) (p = 0.02). Among the six soldiers with positive PCR or ELISA test results, two reported directly caring for COVID-19 patients, four reported having COVID-19 symptoms, and two were isolated for symptomatic SARS-CoV-2 infection that was previously detected by PCR while the soldiers were at JMS. The SARS-CoV-2 infection rate among those who provided direct care to COVID-19 patients was 0.9% (two of 223).

## Discussion

This study of active duty military personnel deployed to care for COVID-19 patients demonstrates that a low rate of SARS-CoV-2 infection among health care personnel in a field hospital is achievable when appropriate resources are coupled with robust infection control measures. Deployed military personnel were from geographic regions with low COVID-19 incidence and were relatively isolated from the community after arriving in NYC. The overall SARS-CoV-2 infection rate among soldiers was <2%, and among those involved in direct patient care, the rate was <1%, which is lower than rates in health care personnel reported in previous studies (*2*–*5*). These findings underscore the importance of use of adequate PPE and rigorous infection control plans for the protection of health care personnel, especially in a field hospital that lacks the standard physical barriers and infrastructure of a traditional health care setting.

JMS protocols and practices highlight the need to ensure compliance with infection control best practices such as assisted donning and doffing of PPE (*6*). Cohorting all COVID-19 patients helped to conserve PPE and reduced the frequency of doffing, thereby limiting risk to medical staff members for exposure to contaminated PPE surfaces during doffing. However, wearing PPE during long shifts can be uncomfortable for staff members. Appropriate monitoring and enforcement can be implemented in any health care setting, but the military command structure is especially well suited for this purpose.

The findings in this report are subject to at least three limitations. First, the patients admitted to JMS were transferred from a hospital in the NYC area with known COVID-19 and were stable or improving before transfer. This resulted in a lower likelihood of these patients being infectious than the typical COVID-19 patient evaluated in a hospital emergency department. Second, the interval between exposure of health care personnel to COVID-19 patients at JMS and serologic testing was relatively short. Because SARS-CoV-2 IgG increases during the first 4 weeks after infection, this short interval reduced the sensitivity of SARS-CoV-2 serologic testing (*7*). Finally, soldiers volunteered to participate and might have had different risk factors and infection rates than did those who did not participate.

The SARS-CoV-2 infection rate among soldiers deployed to NYC was low compared with the rate for other health care personnel cohorts, for both those who cared directly for patients and those who did not. This experience demonstrates that a well-designed, well-resourced infection control plan implemented with fidelity to best practices as well as adequate PPE and isolation of health care personnel from community-based exposures can mitigate the risk for SARS-CoV-2 infection in health care settings, including nontraditional health care settings.

**Summary**

**What is already known about this topic?**

Health care workers caring for patients with COVID-19 are at risk for infection.

**What is added by this report?**

In April 2020, U.S. military personnel were deployed to a New York City field hospital to care for COVID-19 patients. A robust infection control plan was implemented and enforced. Among 336 soldiers participating in an infection risk study, the overall infection rate was 1.7%; the rate among those involved in direct patient care was 0.9%.

**What are the implications for public health practice?**

A well-designed and well-implemented infection control plan and use of adequate personal protective equipment can mitigate the risk for SARS-CoV-2 transmission in health care settings, including nontraditional settings.

## Footnotes

*
https://github.com/nytimes/covid-19-data

†
https://www.thermofisher.com/us/en/home/clinical/clinical-genomics/pathogen-detection-solutions/covid-19-sars-cov-2/interpretive-software.html

§
https://pubmed.ncbi.nlm.nih.gov/33083807/

¶ 45 C.F.R. part 46, 21 C.F.R. part 56; 42 U.S.C. Sect. 241(d); 5 U.S.C. Sect. 552a; 44 U.S.C. Sect. 501 et seq.
